# The Best Interests of the Child from Different Cultural Perspectives: Factors Influencing Judgements of the Quality of Child-Rearing Environment and Construct Validity of the *Best Interests of the Child-Questionnaire* (BIC-Q) in Kosovo and Albania

**DOI:** 10.1007/s12187-018-9543-6

**Published:** 2018-02-22

**Authors:** Daniëlle Zevulun, Wendy J. Post, A. Elianne Zijlstra, Margrite E. Kalverboer, Erik J. Knorth

**Affiliations:** Faculty of Behavioural and Social Sciences, Department of Special Needs Education and Youth Care, University of Groningen, c/o Dr. D. Zevulun, Grote Rozenstraat 38, NL-9712 TJ Groningen, The Netherlands

**Keywords:** Best interests of the child, Cultural perspectives on child-rearing, Migrant children, Return migration, Western Balkans, Mokken scale analysis

## Abstract

Child-rearing practices and beliefs of what determines a ‘good quality’ of child-rearing differ across cultural contexts and more than one interpretation can be given to “a child’s best interests”. This study aims to examine the cultural factors that influence judgements of the quality of children’s rearing environment, and the construct validity of the Best Interests of the Child-Questionnaire (BIC-Q) scale when used in the Western Balkans. In our research on migrant children who returned to Kosovo and Albania, the BIC-Q is used to assess the quality of the child-rearing environment from a local cultural perspective on child-rearing. To assess cultural differences in judgements of the child-rearing environment, we measured agreement through Cohen’s kappa of BIC-Qs completed from a Western-Balkan and a Western-European perspective on child-rearing. The construct validity of the BIC-Q scale was assessed through a Mokken scale analysis. The findings show that – except for two items – there is substantial agreement between Western-European and Western-Balkan assessors regarding the direction of the judgement, *i.e.* if the scores on the child-rearing conditions are dichotomized (sufficient/insufficient). The judgements of the ‘respect’ and ‘interest’ conditions are sensitive to differences in the cultural or professional perspectives of the assessors. The findings of the Mokken scale analysis demonstrate a strong and reliable scale in the cultural context of the Western Balkans (H = .73; Rho = .97). Knowledge gained from using the BIC-Q to assess the living situation of returned migrant children in their countries of origin and insight into child-rearing standards provides input for the best interests of the child determination.

## Introduction

Most children are born into the world with roughly the same endowments and all children need roughly the same basic level of care: “… food, protection, comfort, physical contact, attention, information, guidance and social interaction” (Whiting and Edwards 1988, p. 157). These basic needs are also enshrined in the *International Convention on the Rights of the Child* (CRC 1989), which has been ratified by nearly all countries worldwide. Even though these basic needs can be considered universal for all children, “...the boys and girls of the world enter many different childhoods and depart them through many different doors” (Therborn 2009, p. 338).

The world’s children grow up under different child-rearing regimes or family circumstances (Levine 2007; Levine and New 2008; Weisner 2014; Whiting and Edwards 1988; Whiting and Whiting 1975). The childhood that children experience is determined by the material and ecological setting in which they grow up, the shared values and beliefs in the cultural community, and the practices that foster the necessary skills, behavior and beliefs considered important to assume full membership in the social group (Froerer 2009; Harkness and Super 1996; Weisner 2014). Different child-rearing goals, behaviors and responsibilities for children may thus be expected for those growing up in ‘poor and traditional countries’ compared with those in ‘wealthy and postindustrial countries’ in the Western world (Gielen and Chumachenko 2004).

Due to these cross-cultural differences, more than one interpretations can be given to what is thought to be in “a child’s best interests” (Alston 1994; Freeman 2007), thereby referring to one of the core articles in the CRC (1989). The best interests principle (article 3 CRC) stipulates that in all decisions affecting children, their best interests should be a primary consideration. In General Comment No. 14, the Committee on the Rights of the Child (2013) further elaborates on the elements that should be evaluated and balanced in light of each child’s specific circumstances in such a “best interests of the child determination”. The elements consist of the *individual characteristics* of the child (i.e. child characteristics, views, identity, and vulnerability), and the *social and cultural context* in which children find themselves (i.e. family environment and relations, care protection and safety, right to health and education) (Committee on the Rights of the Child 2013, par. 48–79).

For decision makers taking decisions in cases of migrant children involved in legal procedures regarding their right to stay in (Western) host countries, knowledge of the child’s social and cultural context is indispensable for determining a child’s best interests. Insight into the post-return social-cultural contexts of repatriated migrant children, and knowledge of the local cultural standards regarding child-rearing in countries of origin, could provide decision-makers in host countries with better ‘tools’ for determining children’s interests in migration procedures. Therefore, in a larger European study – the ‘Monitoring Returned Minors’ project – we wished to study the living situation of returnee children after (an enforced or voluntary) return to their countries of origin, Kosovo and Albania. For this purpose, we used the *Best Interests of the Child-Questionnaire* (BIC-Q) (Zijlstra et al. 2012, 2013)

In line with all elements described by the Committee as being essential in a best interests of the child assessment, the BIC-Q analyses the possible scenarios for a child’s development with regard to decisions in migration procedures. The BIC-Q is based on the *Best Interests of the Child Model* (BIC Model) (Kalverboer and Zijlstra 2006; Zijlstra 2012) and adopts a holistic and ecological approach to child development. The BIC-Q consists of seven rearing conditions concerning a child’s *familial* upbringing context (1. adequate physical care; 2. safe direct physical environment; 3. affective atmosphere; 4. supportive, flexible child-rearing structure; 5. adequate examples by parents; 6. interest; 7. continuity in upbringing conditions, future perspective) and seven conditions concerning a child’s *societal* upbringing context (8. safe wider physical environment; 9. respect; 10. social network; 11. education; 12. contact with peers; 13. adequate examples in society; 14. stability in life circumstances, future perspective). Through these 14 conditions, the BIC-Q determines the quality of the child-rearing environment and identifies the risk and protective factors in a child’s upbringing situation.

The BIC-Q child-rearing conditions were selected through a (Western) pedagogical literature review on the criteria for child development within the rearing environment. As is the case with most other psychological research (see also Arnett 2008; Henrich et al. 2010), theories on child development and how to raise children are often constructed in developed Western societies. Therefore, it is most likely that rearing situations of children growing up in non-Western countries – more than 90% of the world’s children (LeVine and New 2008) – are often assessed and judged through a *Western view* on the necessities for a child’s healthy development. A child’s rearing environment could, however, be characterized by circumstances or family situations "…that may appear chaotic and unacceptable by Eurocentric standards, yet they are nonetheless morally and developmentally appropriate in other places" (Weisner 2014, p. 98). When assessing a child’s interests, well-being or living situation in an international context, researchers and decision-makers should thus be aware that the determination of a ‘good quality’ of child-rearing can differ across cultural contexts. The reliability and validity of using Western-developed instruments in a given cultural context should be examined first; simply assuming an instrument to be measuring a ‘universal’ construct may lead to biased results – especially when there are “cultural distances to be bridged” (Van de Vijver and Poortinga 1997, p. 32). Therefore, before using the BIC-Q instrument to measure the quality of the child-rearing environment of returned children in Kosovo and Albania, the *face* and *content validity* of the BIC-Model conditions in the cultural context of the Western Balkans were explored.

### The Child-Rearing Conditions in the Cultural Context of the Western Balkans

The applicability and validity of the 14 BIC-Model child-rearing conditions was assessed through a seminar, an expert’s opinion, and a focus group discussion with Western-Balkan professionals (Zevulun et al. 2015). The participants all considered the conditions of the BIC-Q as being relevant in the Western-Balkan child-rearing environment. However, they indicated that certain cultural aspects affected the meaning and interpretation of the child-rearing conditions in the cultural context of the Western Balkans. These aspects were related to the collectivistic nature of the society, widespread differences within the society, poverty, and the instability as post-conflict states.

First of all, the findings showed that the extended family plays an important role in the daily upbringing of children in the Western Balkans. Apart from parents, also other family members such as grandparents, aunts and uncles may be involved in the daily care and upbringing of children. Second, child-rearing practices are related to an authoritarian child-rearing style, becoming visible in transmitted values such as obedience to and respect for the elderly, and the lower value given to the individual wishes of the child versus the collective interests of the family. Also disciplinary measures such as slapping are still generally accepted, though it seems to be less common compared with the children growing up in the previous generation. Third, the within-society heterogeneity impacts the child-rearing environment for children throughout Western-Balkan societies, such as for children who grow up in rural areas compared with those in urban areas. The children living in rural areas are often involved in assisting in the family livelihood, for instance through farming and tending animals, and parents may be less involved with the emotional needs of their children or available for ‘quality time’. The Roma children, who often live in camps under extremely poor circumstances, are in a particularly marginalized situation compared with their peers belonging to the majority ethnic group. School attendance is not encouraged in all Roma families. Instead, traditional values are often dominant, such as for Roma girls to marry before reaching maturity and for boys providing for family income through child labour. At last, with most Western-Balkan countries still involved in an economic and social transition following the wars and collapse of the communist regimes, the variable stability of the state results in insecure future prospects. As the state is often not involved in child-welfare issues, the extended family and social networks function as a ‘safety net’ and take care of one another when being in need (Zevulun et al. 2015). 

All these aspects permeate the BIC-Model rearing conditions, thereby either influencing the *assessment* (*i.e.*, including extended family members’ child-rearing practices in the assessment) or the *interpretation and meaning* of the child-rearing conditions in the cultural context of the Western Balkans. These findings provided an initial insight into the applicability of the BIC-Q in terms of assessing the quality of the child-rearing environment that children encounter after return to their countries of origin.

### Aim and Research Questions in this Study

This study aims to further analyse the influence of cultural factors on the best interests of the child assessment with the BIC-Q in the cultural context of the Western Balkans, and to study the construct validity of the BIC-Q when completed from a local cultural perspective on child-rearing in migrants’ countries of origin.

The construct validity of the BIC-Q has been previously assessed within the cultural context of Western Europe and completed from a Western-European perspective on child-rearing (Zijlstra et al. 2012). As we aimed to avoid the imposition of a Western-European understanding of a ‘good quality child-rearing’, Western-Balkan professionals assessed the upbringing situation from their cultural perspective on child-rearing. In this study, we analyse the *(dis)agreement in scores* between Western-Balkan and Western-European assessors to identify the cultural factors that could influence the judgement of the child-rearing conditions. We applied a Mokken scale analysis to measure the *strength and reliability* of the scale*.*

Central questions of the study are: 1) whether the Western-Balkan and Western-European assessors score the child-rearing conditions in the same way, and if not, what the differences could be attributed to; and 2) whether the BIC-Q is a reliable and strong scale to measure the quality of the child-rearing environment in migrants’ countries of origin when completed from a local cultural perspective on child-rearing.

## Method

### Sample

In total, 63 families participated in this research, with a majority living in Kosovo (*n* = 49) and a minority in Northern Albania (*n* = 14). We included families who stayed in European Union host countries as migrants or asylum-seekers, returned between 2008 and 2013, and had children between the ages of 11 and 18 years old (re recruitment, see below). Children were included according to an equal distribution of age groups (11–14 years and 15–18 years old), gender, and rural or urban living area.

In Kosovo, we included a group of children belonging to the Roma minority ethnic group as well as children belonging to the Kosovar-Albanian majority ethnic group, as these are the two groups that claim asylum most in the European Union from that region (EASO 2015). In Northern Albania, we included only children from the Albanian ethnic group.

The total sample (*N* = 63) consisted of 35 boys and 28 girls. The children’s ages ranged from 11 to 18 years old, with a median of 13 years (*M* = 13.9; *SD* = 1.96). In Kosovo, 19 children belonged to the Roma ethnic group, and 30 children belonged to the Kosovar-Albanian ethnic group. After their return, 44 children live in an urban area and 19 children in a rural area of Kosovo or Albania. A majority of 42 children and their families returned by force; 21 children and their families returned voluntarily.

The families stayed in 11 European host countries, mostly in Sweden, Belgium, Germany, Italy, and France. The period in which the families applied for the right of residence in the host countries varied between 1990 until 2013. Their duration of stay in the host country ranged from 2 months to 23.2 years, with a median of 3.4 years (*M* = 7.2; *SD* = 6.7).

### Instrument: BIC-Q

With the BIC-Q, the quality of the child-rearing environment can be examined through the sum score of 14 child-rearing conditions in the familial and societal context in which a child develops (see introduction). The scoring categories for qualifying the 14 rearing conditions consist of ‘unsatisfactory’ (0), ‘moderate’ (1), ‘satisfactory’ (2), and ‘good’ (3). When dichotomizing the scoring categories, ‘unsatisfactory’ and ‘moderate’ are computed into *insufficient* (0), and ‘satisfactory’ and ‘good’ into *sufficient* (1).

Research on the reliability and validity of the BIC-Q in a Dutch context from a Western-European perspective (Zijlstra et al. 2012) has shown a good inter-rater and intra-rater reliability (kappas .65 and .74, respectively) and a strong and reliable scale to measure ‘the quality of the child-rearing environment’ (H = .55; Rho = .94). Also, in a Dutch study using records of children living in detention or secure treatment centers, the BIC-Q showed a good inter-rater reliability score (Kalverboer et al. 2012).

### Procedure

The data in this study were collected between June 2013 and December 2014 as part of a European project, which was executed in line with the applicable regulations at that time. Before signing informed consent forms, all children and parents were informed that participation was voluntary, that they could withdraw from the research at any time without having to give an explanation, and that everything shared during the interview would remain confidential and analysed anonymously. All families received ten Euros per child after the interview.

At the time of data collection, no central registration mechanism existed to register returnees in Kosovo or Albania. Municipalities and regional officers in Northern Albania and in all seven districts of Kosovo were approached for their contact information of returnees who returned between 2008 and 2013 from countries in the European Union. Returnees were known to municipalities and regional return offices, for instance because they had registered themselves to obtain assistance after their return. Some lists with contact information of returnees were more up-to-date than others. Some lists contained extensive information with birth dates of the returnees, while others only contained names and telephone numbers. The interviewers contacted all people on the lists to verify whether it concerned a returned family with children.

In Northern Albania, and in one district in Kosovo, children were also recruited through schools. In another district within Kosovo, seven cases were provided by a caseworker who worked for a non-governmental organization that provides assistance to returned families.

The BIC-Q assessment consisted of semi-structured interviews with parents and children and observations of the child-rearing environment. The families were visited and interviewed at their homes by local interviewers (see below).

#### Western-Balkan Perspective on Child-Rearing

The interviews were conducted by four Western-Balkan interviewers working for non-governmental organizations in the field of mental health care and reintegration of returnees in Kosovo. One pair of interviewers with backgrounds in psychiatry and medical sciences conducted 19 interviews, and another pair of interviewers with backgrounds in education, management, and economics conducted 44 interviews.

The interviewer pairs were trained in the BIC-Q assessment by two of the authors (DZ and AEZ). During a field study, a *protocol* was developed in order to ensure uniform practices among the interviewer pairs. *On-the-job training* during the field study was focused on providing detailed clarifications for the scores (Zevulun et al. 2015). The data in this study consist of BIC-Qs that were completed *after* the field study, thus without any outside involvement by the authors.

At the start of the interview, the interview pairs had a conversation with the parents and child together, and talked about factual information related to their migration history and the situation of the family in the host country and after the return. After the joint conversation, one of the interviewers focused on the child and the other interviewer on the parents, and – if possible – interviewed them in separate rooms so that both parents and children had the opportunity to disclose their stories and experiences. Questions were related to the 14 BIC-Q child-rearing conditions and the child’s and family’s life after the return.

After the interviews, which lasted between one to two hours per family, the interviewer pairs scored the 14 conditions according to their local cultural perspective on child-rearing. In the cases that were provided by the caseworker, his view was also included to get a more thorough understanding of the child-rearing environment. In *clarifications* under each BIC-Q condition, the Western-Balkan assessors explained *why* they qualified the rearing environment accordingly.

#### Western-European Perspective on Child-Rearing

An assessor in the Netherlands, who was trained in the assessment with the BIC-Q and has a background in pedagogics, completed the BIC-Qs from a Western-European cultural perspective on child-rearing.

The assessor did not see the family and child, but examined the quality of the child-rearing environment based on 1) the clarifications in the BIC-Qs provided by the Western-Balkan interviewers, without knowing the Western-Balkan judgements of the child-rearing conditions (hereafter referred to as ‘blind BIC-Q’); 2) factual information of the case, for instance the family composition, the return procedure, the Albanian language level of the child, the length of stay in the host country and since the return, and the family’s economic situation after return; 3) the child’s views on the child-rearing situation after return as provided in the self-report version of the BIC-Q (*Best Interests of the Child − Self-report* (BIC-S); Ten Brummelaar et al. 2014, 2017); 4) the child’s responses to social and emotional development items in the *Strength and Difficulties Questionnaire* (SDQ) (Goodman 1997); and 5) in 11 cases, pictures of the living environment of the family after their return. Similar to the Western-Balkan interviewers, the Western-European assessor provided clarifications for each child-rearing condition and explained the qualification of the rearing condition accordingly.

Since the assessment of the quality of the child-rearing environment from the Western-European perspective was based on reports and not on personal contact – and thus differed from the assessment in the previous study on the reliability of the BIC-Q (Zijlstra et al. 2012) – the reliability of the ‘Western-European perspective’ on child-rearing needed to be examined. The reliability was blindly assessed for 29 cases with a second assessor who had the same pedagogical background and experience in assessment with the BIC-Q. In case of poor inter-rater agreement between these two assessors, an expert (AEZ) was asked to provide the decisive judgement on the Western-European perspective, thanks to her thorough experience with the BIC-Q assessment in the Netherlands.

One item (the ‘safe wider physical environment’) seemed to be particularly difficult to assess through the followed procedure, and still showed poor agreement after the expert’s view. Therefore this condition was excluded from the comparison of the Western-Balkan and Western-European qualifications.

### Data Analysis

#### Factors Influencing the Judgement of Child-Rearing Conditions

To study whether cultural factors influenced the judgements of the child-rearing conditions, we compared the Western-European and Western-Balkan scores of the child-rearing environment through Cohen’s kappa. Kappa values were measured for both the general scores (0–3) and the dichotomized scores (0–1). Kappa values above .60 (Cicchetti 1994; Fleiss 1981) and a proportion of agreement above 80% (Feinstein and Cicchetti 1990) were considered as good or excellent agreement between the Western-European and Western-Balkan assessors, and as indicating that no cultural factors influenced the qualifications. Kappa values below .60 indicated a poor or fair agreement between the Western-European and Western-Balkan qualifications.

As there was a procedural difference for completing the Western-Balkan and Western-European BIC-Qs (the Western-European assessors did not see the family and child), we would like to overcome the possibility that variability in scores was caused by this procedural difference. Therefore, we only analysed whether the differences found with regard to the *direction of the judgement* (dichotomized scores ‘sufficient’ or ‘insufficient’ quality) could indeed be attributed to cultural differences*.* In order to know the reasons for the differences in the judgements of the rearing environment, we qualitatively explored the clarifications of the child-rearing conditions that showed kappa values below .60 after dichotomization of the scores.

#### Construct Validity

To examine the construct validity of the BIC-Q (from a Western-Balkan perspective), we analysed whether the 14 child-rearing items in the BIC-Q satisfy the assumptions of a non-parametric item response theory (IRT), using the Mokken scale analysis for polytomous items (Sijtsma and Molenaar 2002).

In the Mokken scale analysis, a *monotonically homogeneous* set of items in the scale assumes: 1) unidimensional latent trait (quality of the child-rearing environment); 2) local independence of the item scores; and 3) monotonicity of item response functions. Hence, the probability of a response on each child-rearing condition as being an increasing function of the quality of the child-rearing environment. An additional assumption of *double monotonicity* means that the item response functions do not intersect. For instance, if a good quality of the ‘adequate physical care’ condition is easier to achieve after return than a good quality of the ‘education’ condition, then, for all returned children, the probability of a positive response on ‘adequate physical care’ is greater than the probability of a positive response on ‘education’. The order of the items is assessed according to the mean scores of the items. A respondent’s position order on the scale is estimated according to the sum score on the 14 items.

We used MSP, a program for Mokken scale analysis for polytomous items (Molenaar and Sijtsma 2000). The ‘test’ option was used for all 14 items with the original codes 0–3. The scalability coefficient *H* for each item, and for the entire scale, was used to determine whether the scale in the cultural context of the Western Balkans satisfied the assumptions of the Mokken model. A value of H < 0.3 indicates a poor scale or item; a value of H between 0.3 and 0.4 indicates a weak scale or item; a value of H between 0.4 and 0.5 indicates a medium strong scale or item; and a value of H > 0.5 indicates a strong scale or item (Molenaar and Sijtsma 2000; Sijtsma and Molenaar 2002).

In order to determine whether the BIC-Q satisfied the double monotonicity assumption, we used the crit values in MSP. Crit values of a certain item exceeding 40 indicate that this model assumption might be violated by the item; a crit value higher than 80 strongly indicates that an item violates the assumption. Due to our small number of cases (*N* = 63), we used a cut-off score of 80 as an indication of violation of the non-intersection assumption (Molenaar and Sijtsma 2000; Sijtsma and Molenaar 2002). If double monotonicity holds, the reliability of the scale can be estimated through the reliability coefficient, rho. A rho >.90 is perceived as excellent (Carmines and Zeller 1994; George and Mallery 2003).

To exclude the possibility of ‘sample dependence’ – *i.e.*, cases where different scales are found for specific subgroups – we conducted a *subgroup analysis* in our sample for the variables age group, gender, return procedure, living area, ethnicity, and country. Due to the small sample sizes of the subgroups, we only focused on the H-coefficient of the scale in these subgroups.

## Results

In this section, we will first describe the results related to the factors influencing the judgements of the child-rearing conditions. Afterwards, the findings are presented on the construct validity of the BIC-Q within the cultural context of the Western Balkans.

### Factors Influencing the Judgement of the Child-Rearing Conditions

Table 1 presents the results of the study into the factors influencing the scores of the child-rearing conditions in the BIC-Q, showing the mean scores, kappa coefficients, and proportion of agreement between the Western-Balkan and the Western-European assessors’ qualifications of the child-rearing conditions in the BIC-Q.

**Table 1:** Mean scores on the child-rearing conditions in the Western-Balkan BIC-Qs, Western-European ‘blind’ BIC-Qs, and agreement between the Western-European and Western-Balkan assessors’ regular and dichotomized scores (*N* = 63)

BIC-Q child-rearing conditions		Mean Western-Balkan BIC-Qs^a^ M (SD)	Mean Western-European ‘blind’ BIC-Qs^a^ M (SD)	Agreement^a^ kappa (proportion agreement)	Agreement dichotomized scores^b^ kappa (proportion agreement)
1.	Adequate physical care	1.68 (1.06)	1.51 (1.32)	.46 (59%)	.84 (92%)
2.	Safe direct physical environment	2.03 (.95)	2.10 (1.09)	.50 (65%)	.67 (86%)
3.	Affective atmosphere	1.98 (.98)	1.78 (1.26)	.54 (67%)	.80 (90%)
4.	Supportive, flexible child-rearing structure	1.71 (1.02)	1.60 (1.21)	.51 (63%)	.71 (86%)
5.	Adequate example by parents	2.13 (.99)	1.71 (1.24)	.38 (56%)	.72 (87%)
6.	Interest	1.70 (1.07)	1.67 (1.22)	.43 (57%)	.55 (78%)
7.	Continuity in upbringing conditions, future perspective	1.76 (1.01)	1.19 (1.28)	.36 (49%)	.75 (87%)
8.	Safe wider physical environment	2.32 (.86)	2.24 (1.04)	–	–
9.	Respect	2.25 (.97)	1.70 (1.12)	.27 (48%)	.49 (78%)
10.	Social network	1.79 (1.08)	1.90 (1.16)	.56 (68%)	.76 (89%)
11.	Education	1.33 (.95)	1.44 (1.07)	.51 (67%)	.81 (90%)
12.	Contact with peers	1.95 (1.20)	1.92 (1.22)	.67 (78%)	.89 (95%)
13.	Adequate examples in society	1.59 (1.03)	1.75 (.97)	.52 (65%)	.81 (90%)
14.	Stability in life circumstances, future perspective	1.51 (1.15)	1.19 (1.28)	.53 (65%)	.77 (89%)

Safe wider physical environment (condition 8) has been excluded from the comparison between Western-Balkan and Western-European BIC-Qs due to inconsistent agreement concerning the Western-European perspective

^a^Scores in BIC-Q: 0 = unsatisfactory; 1 = moderate; 2 = satisfactory; 3 = good

^b^Scores in BIC-Q: 0 = insufficient (unsatisfactory & moderate); 1 = sufficient (satisfactory & good)

The mean scores of the conditions show that the Western-Balkan assessors tend to score the rearing environment as being of a slightly higher quality than the Western-European assessor. For both the Western-Balkan and the Western-European assessors, the mean scores lie around ‘moderate’ and ‘satisfactory’.

For the general BIC-Q scoring categories (0 to 3), the kappa coefficients range from .27 to .67, and the proportion of agreement ranges from 48% to 78%. The outcomes can be mainly qualified as poor or fair agreement, as nearly all conditions have kappa values below .60. The ‘contact with peers’ condition has the highest agreement between the Western-Balkan and Western-European assessors (kappa .67). The ‘adequate examples by parents’, ‘continuity in upbringing conditions’, and ‘respect’ child-rearing conditions have the lowest agreement between the Western-Balkan and Western-European assessors (kappas between .27 and .38).

When dichotomizing the scores into ‘sufficient’ and ‘insufficient’ quality, the agreement between the assessors becomes higher for 11 conditions (kappa between .67 and .89). Hence, no cross-cultural differences are found in the qualifications of these conditions when assessing the quality of the child-rearing environment with regard to the *direction of the judgement*, except for the ‘respect’ (kappa .49; proportion of agreement 78%) and ‘interest’ (kappa .55; proportion of agreement 78%) child-rearing conditions.

In the following section, we will qualitatively explore the clarifications of these two conditions in order to examine whether the differences could indeed be attributed to cultural differences*.*

#### Clarifications for the ‘respect’ Condition

The Western-European assessor qualified the condition ‘respect’ to be of a lower quality (‘unsatisfactory’ or ‘moderate’) than the Western-Balkan assessors in 14 cases. The majority of the cases in which the assessors disagreed concerned Roma children (9 out of the 14 cases).

The assessors both focused on whether the child is discriminated against, accepted in the community and neighbourhood where they live, and whether the child has good contact with relatives and peers. In some cases, the assessors weighed the same information differently – thus, *more negatively* by the Western-European assessor. For instance, in three cases of Roma families, the children are accepted and have good relationships in their neighbourhoods. However, at their schools, the other children do not always welcome them. Another example is that, although in some cases neighbours or relatives support the resettled family, this seems to be out of pity for their situation. Apart from this, the Western-European assessor sometimes considered additional information that was not mentioned by the Western-Balkan assessors with regard to the ‘respect’ condition. These additional considerations focused on whether the child was overlooked and treated unequally in society, for instance, due to not attending school or when no social assistance was provided to their family. In some cases, the child was not leaving the house nor integrating into society.

#### Clarifications for the ‘interest’ Condition

The Western-European assessor qualified the ‘interest’ rearing condition as being of a lower quality (‘unsatisfactory’ or ‘moderate’) than the Western-Balkan assessors in six cases, and as being of a higher quality (‘satisfactory’ or ‘good’) in eight cases.

In the six cases in which the Western-European assessor scored the condition as being of a lower quality, both the Western-Balkan and Western-European assessors focused on the level of parental support of the child, such as assisting with homework, visiting their school, or showing interest and talking with the child about the activities they like to do. However in some cases, due to dynamics within the family, the child was not given much space to voice their interests. It concerns cases, for instance, in which the parents were talking often and openly about stressful topics, such as financial sorrows and the illness of one of the family members. The Western-European assessor weighed these situations *more negatively*. In addition, in two cases the parents stated that they were interested in the child, but that the child was not interested in anything. According to the Western-European assessor, however, these parents did not stimulate their child enough.

In the eight cases in which the Western-European assessor qualified the ‘interest’ condition *more positively* (‘good’ or ‘satisfactory’) than the Western-Balkan assessors, the poor economic situation of the family affected the child’s possibilities to engage in activities of the child’s liking. The Western-European assessor qualified the condition as being of a higher quality when – despite the parents’ financial or other troubles – the parents showed interest in and comforted the child, or felt hopeless that their child could not be involved in any activities due to these troubles.

### Construct Validity BIC-Q

In the Mokken scale analysis (Table 2), the H-coefficient shows that the 14 child-rearing conditions in the BIC-Q form a strong scale to measure the quality of the child-rearing environment in the cultural context of the Western Balkans (H = .73). The reliability of the scale is excellent (Rho = .96). For all separate items, the H-coefficients are strong (H-coefficients between .62 and .82). All conditions have crit values lower than 80 and do not violate the assumption of non-intersection.

**Table 2:** Results of the Mokken scale analysis of the child-rearing items in the Western-Balkan BIC-Qs (N = 63)

BIC-Q Items	Mean score*	H-coefficient per item
Education (11)	1.33	.74
Stability in life circumstances, a future perspective (14)	1.51	.82
Adequate examples in society (13)	1.59	.71
Adequate physical care (1)	1.68	.73
Interest (6)	1.70	.75
Supportive, flexible child-rearing structure (4)	1.71	.80
Continuity in upbringing conditions, a future perspective (7)	1.76	.81
Social network (10)	1.79	.67
Contact with peers (12)	1.95	.71
Affective atmosphere (3)	1.98	.70
Safe direct physical environment (2)	2.03	.71
Adequate examples by parents (5)	2.13	.70
Respect (9)	2.25	.70
Safe wider physical environment (8)	2.32	.62
H = .73; Rho = .96		

*Scores in BIC-Q: 0 = unsatisfactory; 1 = moderate; 2 = satisfactory; 3 = good

Regarding the ranking of the conditions in the scale, the mean scores show that the ‘education’ and ‘stability in life circumstances’ conditions are the *most difficult* rearing conditions to fulfill for the returned migrant children (M = 1.33 and 1.51 resp.). The ‘safe wider physical environment’ and ‘respect’ conditions are more often of a ‘good quality’ after return to Kosovo or Albania (M = 2.32 and 2.25 resp.).

#### Subgroup Analysis

The subgroup analysis in Table 3 shows an overall strong scale for all separate subgroups (H coefficients between .64 to .81) except for children with a Roma ethnicity; here the Mokken analysis shows a medium strong scale (H = .46).

**Table 3:** H-coefficients for the subgroups in the sample

Subgroups	H-coefficient BIC-Q scale
Age group	
• 11–14 years old (*n* = 37)	.64
• 15–18 years old (*n* = 26)	.81
Gender	
• male (*n* = 35)	.75
• female (*n* = 28)	.70
Ethnicity	
• Roma (*n* = 19)	.46
• Albanian (*n* = 44)	.76
Living area	
• rural (n = 19)	.61
• urban (n = 44)	.76
Country	
• Kosovo (n = 49)	.67
• Albania (n = 14)	.66
Return procedure	
• forced return (*n* = 42)	.64
• voluntary return (*n* = 21)	.80

## Discussion

### Factors Influencing the Judgement of the Child-Rearing Condition

One of the aims of this study was to analyse whether cultural factors influenced the best interests of the child assessment with the BIC-Q in Kosovo and Albania. We explored this by focusing on the *judgements* of the child-rearing conditions and comparing the scores on these child-rearing conditions from two different cultural perspectives: a Western-Balkan perspective and a Western-European perspective.

The findings show that there is substantial agreement between Western-European and Western-Balkan assessors when scores on the child-rearing conditions are dichotomized: nearly all conditions show good agreement between the Western-European and Western-Balkan assessors. Only the ‘respect’ and ‘interest’ conditions show a different qualification with regard to the applied perspective to assess the quality of the child-rearing environment (kappas .49 and .55, respectively).

Exploration of the clarifications shows that disagreement on the quality of ‘respect’ for the child might result from a different connotation of this condition across cultures. The Western-European assessor seemed to have a broader and more holistic understanding of ‘respect’ and also focused on whether the child lived isolated from society and peers. The Western-European assessor often qualified the condition as being of a lower quality when the state or wider society paid little attention to the child, even if the child was in a vulnerable situation. This difference might stem from a higher state involvement in children’s welfare issues in Western-European states, while in Western-Balkan countries this is less institutionalized (Zevulun et al. 2015).

The connotation of ‘respect in a societal context’ might also be dependent on how ‘the child’ is viewed in individualistic and collectivistic societies. Children growing up in developing (often collectivistic) societies are expected to contribute to the family household from a young age onwards, with roles assigned to them by their gender: for instance, boys may be expected to assist in farming or other types of work, while the girls do domestic chores and care for their siblings (Kostelny 2006). The children in these societies are often educated with values focused on religious piety, conformity, interdependency, social cohesion and loyalty to the family or group, while children in developed (often individualistic) societies are raised with values directed towards their autonomy, self-actualization and independency, and increasing egalitarianism regarding gender roles (Gielen and Chumachenko 2004, p. 103; Kağitçibaşi 2006). Thus, while in the Western (‘individualistic’) context the child is viewed as “… an autonomous entity or as developing into one”, in collectivistic societies children are more often viewed as part of the wider social group or family, and have “…collective identities that honor the good of the group over any individual good” (Kostelny 2006, p. 21). Therefore, in collectivistic societies such as exists in Kosovo and Albania, the ‘respect’ child-rearing condition might not only be focused on the child as an individual but even more so on the child as a member of the (family) group.

Another explanation regarding disagreement on the quality of ‘respect’ in cases of Roma children might be related to be the assessors’ experiences with *inter-ethnic relations* in society. The Roma population in Kosovo generally lives under segregated and poor living conditions, and especially the forced returnees seem to be marginalized due to discrimination, problems repossessing property and difficult access to education, health and social welfare services (Human Rights Watch 2010). The Western-Balkan assessors acknowledged also in other conditions that the position of the Roma is generally low in Kosovar society. The ‘respect’ condition seemed to be of sufficient quality when the child was accepted in the neighbourhood and had good contacts with relatives, neighbours, or peers. However, according to the Western-European assessor, such circumstances were not always satisfactory – for instance, when the wider population, especially at school, did not accept the child or when neighbours and relatives only seemed to support the family because they pitied them.

The examination of the clarifications for ‘interest’ shows that the Western-European assessor evaluated primarily the caregivers’ provision of space for the child to express wishes and activities of the child’s liking, while the Western-Balkan assessors mainly focused on whether or not the child is given opportunities to conduct activities of the child’s liking (*e.g.*, due to financial difficulties). Hence, the Western-European assessor seemed to apply a more *pedagogical approach* to the ‘interest’ condition. This might be a result of the differing professional backgrounds of the assessors. Further inspection of the data showed that regarding the ‘interest’ condition the Western-European assessor disagreed more often with the interviewer pair having a management and economics background (*n* = 13; 29% of their cases), than with the interviewer pair having a background in psychiatry and medical sciences (n = 1; 5% of their cases). On the other hand, this disagreement did not occur with regard to other conditions that are focused on the parenting style.

We cannot determine whether the disagreement regarding the ‘interest’ condition was related to the assessors or to cultural differences regarding child-rearing. Future research should provide more insight into whether these differences could indeed be attributed to differences in *cultural perspective* and/or in *professional backgrounds* of assessors.

### Construct Validity

The aim of the Mokken scale analysis was to examine the construct validity of the BIC-Q when used in another cultural context, and completed from another cultural perspective, than the Western context in which it was developed. There were no problematic items in the scale. The findings of the Mokken scale analysis show that the BIC-Q is a strong scale to measure the quality of the child-rearing environment in the cultural context of the Western Balkans (H = .73; Rho = .96).

The scale seems to be strong for most subgroups in the sample, except for Roma children (H = .46). We are not certain why the Mokken analysis shows a medium strong scale for the Roma children in our sample. In our previous study (Zevulun et al. 2015)*,* we assessed the content validity of the scale in the cultural context of the Western Balkans based on broad patterns of family life, kinship relations, and characteristics of Western-Balkan societies (see also Goodman 2004). Only regarding the ‘education’ condition were different values found for children growing up in Roma families; we did not have other indications that the BIC-Model rearing conditions were not relevant for child-rearing in Roma families. The assessment of the quality of the child-rearing environment and applicability of the BIC-Q in Roma families should be subject of future research.

The construct validity seems to be sufficient for Western-European (Zijlstra et al. 2012) and Western-Balkan perspectives (this study). The ordering, however, is different for both scales, suggesting sample dependence between migrant children in *the host country* and migrant children *after return*. The ranking of the items in the Mokken scale shows that ‘stability in life circumstances’ and ‘education’ are the *most difficult* items in which to obtain a good score for migrant children after return. In the previous study into the construct validity of the BIC-Q from a Western-European perspective (Zijlstra et al. 2012), not ‘education’ but ‘stability in life circumstances’ and ‘continuity in upbringing conditions’ were the most difficult conditions for the children during the stay in the host country.

These two conditions measure the child-rearing environment in the familial and societal context over a longer period of time and are of great importance: if environmental conditions are of insufficient quality during a longer period this might have developmental consequences for a child (Zijlstra 2012). Experiencing instability is eminently present in migrant children’s lives; migrant children and their families face different kinds of transitional experiences, as they need to move often and adjust to new living environments (Fong 2004; Suárez-Orozco and Suárez-Orozco 2002). For returned migrant children, the instability can have an additional impact – especially for the children who were fully integrated in the host country and lacked any concrete memories of living in the country of origin (Cornish et al. 1999; Knaus et al. 2012; Kalverboer et al. 2009; Riiskjaer and Nielsson 2008). The condition ‘continuity in0 upbringing conditions’, however, was an easier condition to fulfil after return than during the stay in the host country. A possible explanation might be that the returnee children re-established bonds with ‘significant persons from their past’, such as grandparents and other extended family members.

Regarding the other item that seems to be difficult after return – ‘education’ – the returned children faced an additional transition. It seems to be difficult for the children to join in with the education of their peers in the countries of origin. In our previous study (Zevulun et al. 2015), the specific impact of ‘local standards’ on the returned children was especially noted in education. Not only did the language barrier make it more difficult to reintegrate, but also the different values and disciplinary styles of teachers had an additional negative impact on the returned children. Another explanation for the generally low quality of the ‘education’ condition in the Kosovar and Albanian context may be that the Western-Balkan assessors considered education a very important condition in children’s upbringing. The local standards in terms of scoring this condition had been the subject of discussion among the interviewer pairs (Zevulun et al. 2015). Hence, the weight and importance attached to the ‘education’ condition within a best interests of the child assessment might be different when completed from another cultural perspective (see also Fuligni 2004; Xu 2014).

The ‘safe wider physical environment’ and ‘respect’ conditions were most often of a good quality after the return in Kosovo and Albania. As countries in the Western Balkans are considered as ‘safe countries of origin’ by many European Union member states (EPRS 2015), these BIC-Q conditions are likely to be more difficult in countries where returnees face security issues or persecution after their return (Carr 2014; Davids and Van Houte 2008; Bowerman 2017).

### Strengths and Limitations

Cross-cultural differences in child-rearing practices and beliefs can lead to different interpretations of what is considered in a child’s best interests (Alston 1994; Freeman 2007), and necessitate validation of instruments that are based on Western theories of child development before using them in another cultural context (Van de Vijver and Poortinga 1997). Through this study we were able to provide further insight into the cultural factors affecting the judgement of the social and cultural context in which children find themselves after repatriation, and into the construct validity of the BIC-Q scale when used in migrants’ countries of origin and completed from a local cultural perspective.

A limitation of the study into the factors influencing the judgement of the conditions is a procedural difference; the Western-European assessor based the judgement of the rearing environment on the clarifications of the Western-Balkan assessors and did not have access to all the interview material and observations of the rearing environment. Being ‘cultural insiders’, the local interviewers might have had pre-constructed assumptions and knowledge about how things were organized in the region where they live, a result of which being the possibility they may have taken for granted specific situations and child-rearing practices in their clarifications (Kanuha 2000; Labaree 2002). On the other hand, we sought to overcome this possible bias through on-the-job training during the previous field study, focusing on continuous reflection, and writing detailed clarifications that were understandable for the assessors in the Netherlands (Zevulun et al. 2015).

Within the framework of this study, it was not possible for the Western-European assessor to interview the parents and children as well, and to complete the BIC-Q through exactly the same procedure as the Western-Balkan assessors did. To overcome the possibility of variability in scores being caused by the procedural difference, we only analysed whether the disagreement regarding the direction of the judgement (sufficient/insufficient) could be attributed to the cultural differences in child-rearing.

Another limitation concerns the relatively small research sample for the Mokken scale analysis. Tracing returnee children in Kosovo and Albania was complicated as there was no central registration mechanism enlisting all returned families. Instead, we only had access to those cases that were registered at municipalities. Families who did not approach the authorities were thus not included: possibly left out of the sample were those families not in need of assistance or those who did not contact authorities due to issues of mistrust or plans to re-migrate. In Albania and one municipality in Kosovo, cases had to be recruited through schools. Therefore, in our Albanian research group, nearly all the children are going to school, while studies show that returned children in Kosovo and Albania can face difficulties regarding school enrolment (Knaus et al. 2012; UNICEF 2015). Thus, in these two districts, we may have missed returned children who were not enrolled in schools or who were otherwise ‘invisible’ to the authorities. Nevertheless, as our subgroup analysis showed a medium strong to strong scale for the different subgroups in our sample (H coefficient between .46 and .81), we have no indications that a different sample would yield different findings on the internal consistency and strength of the scale. Future research should provide more insight into the sample independence of the BIC-Q scale when used in an international context.

### Implications for Research and Practice

The Committee on the Rights of the Child states that in every decision affecting a child, the child’s best interests should be assessed through analysing the possible scenarios for a child’s development in the short and long term (Committee on the Rights of the Child 2013, par. 84). In order to secure the child’s best opportunities for development after (forced or voluntary) return to a country of origin, assessment with the BIC-Q can give input on the risk and protective factors in the child’s social and cultural context, and could provide indications as to how the child should be supported or prepared for the return. This study pointed towards some differences in the judgment of the child-rearing environment when the instrument was completed from different cultural perspectives. In order to be used as a ‘universally-valid’ instrument in the return decision-making and for the preparation of migrant children needing to return to their country of origin (Kalverboer 2014), additional research steps should be taken.

First of all, the factors influencing the qualifications of the BIC-Q conditions were assessed from a Western-Balkan and a Western-European cultural perspective. Despite large differences between rural and urban areas in Kosovo, especially the young generation in Kosovo seems to regard itself as part of Western Europe (Jones et al. 2003). As assessment in the current study shows “…how the returned children are faring according to an ‘urban’ perspective of the majority population group in Kosovo” (Zevulun et al. 2015, p. 514),  it might be possible that the Western-Balkan and Western-European assessors did not differ that much from one another with respect to cultural values and norms related to child-rearing. Therefore, it would be interesting for future studies to identify which child-rearing conditions in the BIC Model seem to be ‘universally’ valid and which ones ‘culturally dependent’ when the instrument is applied in cultural settings with larger cultural distances. In addition, the findings showed that differences in qualifications of the rearing environment could have resulted not only from cultural differences, but also from the professional backgrounds of the assessors who completed the instrument. Future studies should aim to conduct this kind of cross-cultural study with assessors having the same professional backgrounds – preferably consisting of a multi-disciplinary team (Committee on the Rights of the Child 2013, par. 47) – and having access to the same data.

As this study suggested sample dependence by the different ranking of the items for children in the host country (Zijlstra et al. 2012) and for children after return (this study), it would be interesting for future studies to analyse more deeply why differences in the item ranking occurred. Such an analysis can identify which child-rearing conditions and opportunities for development are particularly subject to change after repatriation, and provide additional evidence on the cultural sensitivity of the BIC-Q items; if a certain item is more easy or difficult than another item for one culture but not for the other, the item might also tap a different attribute across cultures (Hui and Triandis 1985).

An implication for practice relates to the specific research group of migrant children and the determination of their best interests in accordance with article 3 CRC. Migrant children may have faced adverse experiences during their lives before their migration, during their migration, and during their stay in the host country (Bronstein and Montgomery 2011; Fazel et al. 2012; Van Os et al. 2016). These circumstances may negatively influence the vulnerability of the child and exacerbate the impact of insufficient child-rearing conditions in a child’s life. Furthermore, due to their forced movements and integration in different cultural contexts, migrant children are acquainted with differences in upbringing situations across countries. Dependent on their length of stay in the host country, they often adapt to the host countries’ cultural practices, norms, and values and become rooted there (Kalverboer et al. 2009). Therefore, in our opinion, the child’s vulnerability and ‘personal fit’ with cultural practices in the country of origin should be established and taken into account *before* deciding on the cultural standards through which a child’s best interests are determined. It concerns an ethical consideration that should be taken into account when determining children’s best interests with regard to return to the country of origin.
